# Listener characteristics differentially affect self-reported and physiological measures of effort associated with two challenging listening conditions

**DOI:** 10.3758/s13414-020-02195-9

**Published:** 2021-01-12

**Authors:** Alexander L. Francis, Tessa Bent, Jennifer Schumaker, Jordan Love, Noah Silbert

**Affiliations:** 1 Department of Speech, Language and Hearing Sciences, Purdue University, Lyles-Porter Hall, 715 Clinic Dr., West Lafayette, IN 47907 USA; 2 Department of Speech, Language and Hearing Sciences, Indiana University, Bloomington, IN USA; 3 Applied Research Laboratory for Intelligence and Security, University of Maryland, College Park, MD USA

**Keywords:** Cognitive and attentional control, Physiological psychology, Speech perception

## Abstract

Listeners vary in their ability to understand speech in adverse conditions. Differences in both cognitive and linguistic capacities play a role, but increasing evidence suggests that such factors may contribute differentially depending on the listening challenge. Here, we used multilevel modeling to evaluate contributions of individual differences in age, hearing thresholds, vocabulary, selective attention, working memory capacity, personality traits, and noise sensitivity to variability in measures of comprehension and listening effort in two listening conditions. A total of 35 participants completed a battery of cognitive and linguistic tests as well as a spoken story comprehension task using (1) native-accented English speech masked by speech-shaped noise and (2) nonnative accented English speech without masking. Masker levels were adjusted individually to ensure each participant would show (close to) equivalent word recognition performance across the two conditions. Dependent measures included comprehension tests results, self-rated effort, and electrodermal, cardiovascular, and facial electromyographic measures associated with listening effort. Results showed varied patterns of responsivity across different dependent measures as well as across listening conditions. In particular, results suggested that working memory capacity may play a greater role in the comprehension of nonnative accented speech than noise-masked speech, while hearing acuity and personality may have a stronger influence on physiological responses affected by demands of understanding speech in noise. Furthermore, electrodermal measures may be more strongly affected by affective response to noise-related interference while cardiovascular responses may be more strongly affected by demands on working memory and lexical access.

In ideal contexts, for listeners with good hearing, understanding spoken language often seems effortless and automatic. However, listening conditions are seldom optimal outside the laboratory or clinic. In particular, there may be noise in the background and talkers may be hard to understand because their speech differs from expectations—for example, due to a nonnative accent (Van Engen & Peelle, 2014). The ability to understand speech under adverse listening conditions varies widely across listeners, even for young adult listeners with normal hearing (e.g., Benichov, Cox, Tun, & Wingfield, 2012; Bent, Baese-Berk, Borrie, & McKee, 2016; McLaughlin, Bent, Baese-Berk, Borrie & Van Engen, 2018; Wightman, Kistler, & O’Bryan, 2010). However, the underlying basis for such differences in performance is not yet well understood. While there is relatively strong evidence that cognitive and linguistic capacities play a role, there is also increasing evidence that such factors may not contribute equally to success in all adverse conditions.

Individual differences in listening performance under adverse conditions may derive from many different sources. There is a broad consensus that listeners faced with noise-related or speech-related listening challenges must allocate cognitive resources to overcome them (Pichora-Fuller et al., 2016; Shinn-Cunningham & Best, 2008; Van Engen & Peelle, 2014; Zekveld, Kramer, & Festen, 2011). Likely resources (broadly defined) include working memory capacity (Brännström, Karlsson, Waechter, & Kastberg, 2018; Ingvalson, Lansford, Fedorova, & Fernandez, 2017a, 2017b; Pichora-Fuller et al., 2016; Rudner, Lunner, Behrens, Thorén, & Rönnberg, 2012; Wingfield, 2016), selective attention/executive control (Heald & Nusbaum, 2014; Song & Iverson, 2018; Strauss & Francis, 2017; Ward, Shen, Souza, & Grieco-Calub, 2017; Wild et al., 2012), processing speed (Ingvalson et al., 2017a), and/or other executive functions such as inhibitory control and switching cost (Brännström et al., 2018; Ingvalson et al., 2017a, 2017b; Perrone-Bertolotti et al., 2017). Thus, individual differences in cognitive capacity, perhaps especially selective attention and working memory, may underlie differences in listening performance both within and across contexts.

Similarly, a variety of linguistic factors have been identified as possible contributors to individual differences in speech perception under adverse listening conditions. Specifically, listeners with larger vocabularies tend to be more accurate in perception of native speech in noise (McLaughlin et al., 2018; Tamati, Gilbert, & Pisoni, 2013), dysarthric speech (Bent et al., 2016; Ingvalson et al., 2017b; McAuliffe, Gibson, Kerr, Anderson, & LaShell, 2013), naturally produced unfamiliar accents (Bent et al., 2016; McLaughlin et al., 2018), and a constructed unfamiliar accent (Banks, Gowen, Munro, & Adank, 2015), as well as for adaptation to an unfamiliar constructed accent (Janse & Adank, 2012; however, see Benichov et al., 2012, for conflicting results). Further, an enhanced ability to use indexical properties (e.g., regional dialect or nonnative accent identification) is correlated with word recognition in noise (Atagi and Bent, 2016; Tamati et al., 2013). Thus, these studies highlight the importance of individual differences in linguistic capabilities, especially those related to vocabulary.

## Two approaches to investigating individual differences

Studies linking individual differences in cognitive and linguistic capacities or traits to speech recognition under adverse conditions generally suggest a role for working memory capacity and/or linguistic capabilities (Akeroyd, 2008; Besser, Koelewijn, Zekveld, Kramer, & Festen, 2013; Tamati et al., 2013). These studies typically correlate performance on different cognitive or linguistic tasks with speech recognition performance, which we therefore will call a trait-correlational approach. However, the effects of working memory capacity, in particular, may be weaker in younger adults and the type of task used may also play a significant role in the strength of any correspondence observed (Füllgrabe & Rosen, 2016a, 2016b).

Similarly, studies comparing individual listeners’ performance across different challenging listening conditions suggest that listeners may differ in the degree to which their specific strategies are successful in a given condition. We refer to studies comparing measures from different conditions as a “task comparison” approach. Previous uses of this approach have proposed that measures may differ within participants but across tasks either because different listeners employ different strategies in a given condition (Bent et al., 2016) or because they employ similar cognitive strategies across varying adverse conditions but with differing degrees of success across individuals (Borrie, Baese-Berk, Van Engen, & Bent, 2017). Finally, McLaughlin et al. (2018) combine these two accounts, arguing that listeners may apply some cognitive resources or strategies more generally (i.e., those involving lexical access) while other resources are engaged in a source-specific or environment-specific manner.

Here, we use multilevel modeling to simultaneously investigate the role of both individual differences in a priori measures of cognitive and linguistic faculties (i.e., the trait-correlational approach), while also examining individual differences in responsivity in two different effortful listening conditions (i.e., the task comparison approach). We also attempt to match the difficulty of major aspects of the two conditions for each participant, such that individual differences in performance across conditions are likely to be represented as only minor asymmetries within the same rough range of scores. Constraining the two conditions to roughly equivalent performance ranges also facilitates the assessment of listening effort, a dependent measure that may provide additional insight into listeners’ abilities to cope with challenging listening conditions (Pichora-Fuller et al., 2016).

## Listening effort

Research using a trait-correlational approach has shown a relationship between speech perception in adverse conditions and individual differences in cognitive and/or linguistic capacities. However, the *effortfulness hypothesis* (Lunner, Rudner, & Rönnberg, 2009; McCoy et al., 2005; Pichora-Fuller, Schneider, & Daneman, 1995; Pichora-Fuller & Singh, 2006; Rabbitt, 1968, 1991; Surprenant, 2007; Tun, McCoy, & Wingfield, 2009; Wingfield et al., 2005) suggests in addition that useful correspondences may potentially be found by examining measures related to cognitive effort instead of or in addition to performance. According to this hypothesis, as a task becomes more challenging (noise becomes louder, the acoustic-phonetic properties of an unfamiliar accent diverge more from what is expected), more cognitive resources must be devoted to processing to maintain performance. The deployment of these resources can be characterized as effort (see especially Pichora-Fuller et al., 2016) and may be quantified in terms of a variety of behavioral and physiological measurements (see reviews, including concerns about what, exactly, constitutes listening effort, by Francis and Love, 2020; McGarrigle et al., 2014). Once again, different conditions may incur demand on different resources and to varying degrees, and thus may result in differences in measured effort even when performance is comparable (Alhanbali, Dawes, Millman, & Munro, 2019; Lau, Hicks, Kroll, & Zupancic, 2019; Strand, Brown, Merchant, Brown, & Smith, 2018).

Here, we focus on psychophysiological measures of listening effort that have the advantage that they may be conducted unobtrusively (i.e., without interrupting the listening task, and without depending on post-task recollection of effort. When perceiving speech in adverse conditions, allocation of cognitive resources may induce physiological, especially autonomic, responses (Cvijanović, Kechichian, Janse, & Kohlrausch, 2017; Francis, MacPherson, Chandrasekaran, & Alvar, 2016; Koelewijn, Shinn-Cunningham, Zekveld, & Kramer, 2014; Kramer, Kapteyn, Festen, & Kuik, 1997; Kuchinsky et al., 2013; Mackersie & Calderon-Moultrie 2016; Mackersie & Cones 2011; Seeman & Sims, 2015; Winn, Edwards, & Litovsky, 2015; Zekveld et al., 2011; see review by Francis & Oliver, 2018). However, recent explicit comparisons measuring listening effort resulting from different kinds of signal manipulation and employing different but simultaneous dependent measures of effort have shown little or no correlation across measures (Alhanbali et al., 2019; Lau et al., 2019; Strand et al., 2018). For example, Alhanbali et al. (2019) showed that a suite of behavioral and physiological responses often used as measures of listening effort, including measures of pupil dilation, electrodermal activity (EDA), and EEG, were only weakly correlated with one another despite showing good reliability individually. Similarly, Lau et al. (2019) found no significant correlations between subjective (self-report) and physiological (pupil dilation) measures of listening effort across six conditions, while Strand et al. (2018) found only relatively weak correlations between different measures of listening effort including self-report, dual-task, and physiological (pupil dilation) measures. In all cases, this general lack of strong correspondence between separate measures of listening effort was interpreted as suggesting that different measures may reflect different sources of, or cognitive responses to, increased listening demand (see also discussion by Francis & Oliver, 2018; Strauss & Francis, 2017).

## Individual differences in listening effort

A number of studies have attempted to identify individual differences in cognitive capacities or other behavioral traits that might help to resolve the observed lack of correlation between different measures of listening effort. One consideration underlying such studies is the possibility that physiological measures may be differentially associated with the application of specific cognitive mechanisms, and therefore individual differences in the ability to apply those mechanisms (i.e., capacity limitations) may explain individual differences in patterns of effort measurements. For example, Strand et al. (2018) examined the relationship between listening effort and a variety of cognitive and personality measures, and found a general correspondence between higher cognitive capacity (based on tests of lexical decision, working memory, listening span, and inhibition, among others) and performance on tasks associated with listening effort (repetition of spoken sentences presented in noise). However, examination of the interaction between the cognitive measures and *changes* in demanded listening effort resulting from changes in signal-to-noise ratio (SNR) showed few significant interactions and those that were significant showed divergent patterns of dependency. Strand et al. (2018) interpreted these results as suggesting that different challenges to intelligibility (e.g., noise masking vs. unfamiliar accents) may interact with the demands of a given task (e.g., sentence repetition). They also proposed that individuals may differ in the degree to which they can overcome listening challenges brought about by the interaction between demands imposed by specific speech stimuli and tasks. Our goal in the present work is to investigate this possibility by evaluating individual differences in measures of listening effort across two different listening contexts (noise masking vs. nonnative accent) that are comparably difficult.

In this study, we include a variety of measures of individual differences previously shown to have an influence on speech recognition and/or listening effort, including age (Ingvalson et al., 2017a, 2017b), hearing status (Ingvalson et al., 2017a, 2017b), vocabulary (McLaughlin et al., 2018; Tamati et al., 2013), and working memory capacity (Akeroyd, 2008; Brännström et al., 2018). We also include a measure of selective attention based on the work of Dalton and Lavie (2004), a brief measure of personality traits (John & Srivastava, 1999; Rammstedt & John, 2007), a measure of noise sensitivity (Schutte, Marks, Wenning, & Griefahn, 2007), and (because we set stimulus levels individually), measures of signal levels for each participant.

## Physiological measures associated with listening effort

In addition to self-reported measures of listening effort, we also include measures of arousal and valence that have previously been associated with cognitive, especially listening, effort. These are (1) electrodermal activity (EDA), reflecting changes in skin conductance due to increasing or decreasing eccrine sweat-gland activity (Andreassi, 2007; Boucsein, 1992; Dawson, Schell, & Filion, 2007). Increased skin conductance level (SCL) has been associated with increased demand on working memory (e.g., in an *N*-back task; Mehler, Reimer & Coughlin, 2012), and decreases in SCL are associated with decreases in sustained attention (vigilance; Davies & Krkovic, 1965), suggesting that SCL may serve as a strong indicator of cognitive engagement in a task, perhaps especially one involving demand on working memory. (2) The prevalence of nonspecific (also called spontaneous) skin conductance responses (SCRs). SCRs are transient increases of skin conductance over a relatively short period of time (1–63 s) that have been associated with the engagement of working memory and/or selective attention, especially with respect to emotionally salient stimuli (Andreassi, 2007; Bradley, 2009; Cohen, 2014) and speech recognition (Francis et al., 2016; Mackersie & Calderon-Moultrie 2016; Mackersie & Cones 2011; Seeman & Sims, 2015; though see Cvijanović et al., 2017; Mackersie, MacPhee, & Heldt, 2015, for null results). (3) The frequency or period of the heartbeat is measured because a broad range of studies have shown increased heart rate (decreased heart period) in more cognitively demanding task conditions (Backs & Seljos, 1994; Carroll, Phillips, & Bolanos, 2009; Carroll, Turner, & Prasad, 1986; Kennedy & Scholey, 2000; Turner & Carroll, 1985), including in one study of listening effort (Seeman & Sims, 2015), though not in others (Cvijanović et al., 2017; Mackersie & Cones, 2011). Here we report measures of heart period, not heart rate, because they more directly relate to autonomic arousal when comparing across individuals with different baseline heart rates (see Discussion and references to experimental data by Berntson, Cacioppo, & Quigley, 1995). (4) The amplitude of the blood volume pulse measured in the capillary bed of the hands or feet has also been shown to correspond to increasing demand from the Stroop task (Tulen, Moleman, Van-Steenis, & Boomsma, 1989), mental arithmetic (Goldstein & Edelberg, 1997), and increased working memory load (Iani, Gopher, & Lavie, 2004). And finally, (5) facial electromyography (EMG) is one of the predominant physiological measures for assessing affective valence (Cacioppo, Petty, Losch, & Kim, 1986; Potter & Bolls, 2012), and had been used successfully in the study of listening effort. For example, Mackersie and Cones (2011) found a significant relationship between task difficulty in a dichotic digits task and activity in the frontalis muscle (indicating some degree of brow-furrowing), suggesting that task demands differentially affected activity in this muscle. Here, we examine activity in corrugator supercilii, because not only does increased corrugator activity reliably indicate negative affect (i.e., displeasure, anger, frustration), but relaxation of corrugator may also indicate a pleasurable (i.e., positive affective) response (Larsen, Norris, & Cacioppo, 2003; Potter & Bolls, 2012).

We focus on tonic rather than phasic measures because tonic responses may be more representative of the longer-term reactions evoked in natural listening contexts (cf. Winn & Moore, 2018), but we examine these measures only while listeners were attending to story-length stimuli rather than under even more naturalistic conversational conditions (e.g., Cvijanović et al., 2017), to avoid introducing variability in physiological measurements due to speech and other physical movement during data acquisition.

## Summary

The present study was designed to explore how individual differences, especially in cognitive and linguistic capacities, might relate to performance and listening effort in two different challenging listening conditions. Our goal was to determine whether some of the widely reported individual variability in response (performance or listening effort) to different challenging listening conditions might be attributable to individual differences in cognitive or linguistic capacities, or other demographic properties such as age, hearing status, or noise sensitivity. Here, we investigate individual differences in spoken language comprehension and listening effort under two different challenging listening conditions, one involving noise-masked native-accented English speech and the other consisting of nonnative accented English speech without masking.

## Method

### Overview

The overall structure of the present study involved first collecting data to quantify *individual differences* characterizing each participant’s working memory capacity, selective attention, hearing acuity, receptive vocabulary, personality traits, and noise sensitivity. Participants then performed two *word recognition* tasks: one with native accented speech presented in noise (henceforth, the *noise* condition) and one with a nonnative speaker (henceforth, the *accented* condition). An estimated signal-to-noise ratio (SNR) was calculated to equate each listener’s word recognition in noise performance to their word recognition for the nonnative accented talker. This step was performed in an attempt to minimize performance differences across the two conditions. Finally, listeners completed the main *story comprehension task* in which they heard a series of short stories presented in noise by a native talker and a matched set of stories produced by a nonnative, accented talker and answered multiple-choice questions about them. Story comprehension performance and physiological and self-reported measures of listening effort were collected during the story listening task.

### Participants

Forty-eight participants (ages 18–73 years, 19 males, 29 females) were recruited for this study. Participants were screened for anxiety, depression, and cognitive ability using three standard instruments: The Geriatric Anxiety Index (GAI; Pachana et al., 2007), the Geriatric Depression Scale short form (GDS; Yesavage & Sheikh, 1986), and the Montreal Cognitive Assessment (MoCA; Nasreddine et al., 2005), respectively (see Table 1 for inclusion thresholds). Pure-tone hearing thresholds were acquired for each ear (see below) and participants completed an extensive survey of health-related characteristics. Based on these screeners, data from a total of 35 participants were retained for analysis. Demographic information for the 35 analyzed participants is shown in Table 1. Nine of the 13 excluded participants were removed for the following reasons: (a) Pure-tone hearing thresholds in both ears above the 95% confidence interval reported by Lee et al. (2012) at any of 500, 1000, 2000, or 4000 Hz (four participants, ages 20 [F], 48 [M], 70 [F], and 73 [M]); (b) scored more than one standard deviation below the mean for their age group on the MoCA (see below), as reported by Borland et al. (2017) (one participant, age 39 [F]); (c) did not complete both sessions of the study (two participants, both male, ages not reported); and (d) reported significant prior experience with Chinese-accented English during debriefing (two participants, 25 [F] and 18 [M]).

**Table 1:** Inclusion-related variables for participants in the final analysis group

	Age	GAI	GDS	MoCA
Threshold for inclusion	18+	<10	<6	Age < 65: ≥ 26 Age 65+: ≥ 25
Mean	37.14	2.11	1.17	28.37
Median	27.00	1.00	1	28
Minimum	18	0	0	26
Maximum	71	9	5	30
IQR	25.5	3	2	1

*Note.* Age = age in years; GAI = score on the Geriatric Anxiety Index (Pachana et al., 2007); GDS = score on the Geriatric Depression Scale (Yesavage & Sheikh, 1986); MoCA = composite score on the Montreal Cognitive Assessment test (Nasreddine et al., 2005); IQR = interquartile range

In addition to these nine participants, four were excluded for currently taking medication known to affect broad autonomic nervous system function (ages 43 [F], 62 [M], 66 [M], 71 [F], see the following). Although participants were screened for current medication usage that might affect behavioral or autonomic response and anyone taking stimulant medication (i.e., for ADHD) was excluded prior to participation. Additional medication usage was noted at the time of testing, but only examined once the final data set was collected. Data from participants taking statins or beta blockers for high blood pressure were excluded completely (*N* = 3), as were data from one participant taking Bentyl (an antispasmodic that may affect electrodermal and other autonomic responses related to smooth muscle activity). Beyond these four who were completely excluded, data from participants taking angiotensin II receptor blockers (*N* = 1), diuretics (*N* = 1) or ACE inhibitors (*N* = 1) for blood pressure were ultimately retained, as were those taking antidepressants (*N* = 2). However, physiological data involving vascular dilation (blood volume pulse amplitude [BVPA] measures) from the participant taking ACE inhibitors were not analyzed.

Recruitment was limited to nonsmokers or former smokers. Of the included participants, seven had previously smoked but had quit between 1 and 27 years prior to participation. Five participants reported never consuming caffeine; eight did not drink alcohol. No participant reported having consumed recreational drugs in the past 24 hours.

### Materials and stimuli

#### Individual differences measures

##### Working memory capacity

To assess working memory capacity, participants completed an auditory *N*-back task (Kirchner, 1958; see Redick & Lindsey, 2013). Stimuli consisted of the spoken English letters *A, D, H, J, M, O, Q, R, S,* and *Y*. These were chosen by identifying maximally distinct items in Walker’s (1989) analysis of Hull’s (1973) confusion matrices. Stimuli were produced by a 23-year-old female native speaker of English and were recorded in a double-walled sound booth (IAC Inc.) on a Marantz PMD 660 digital audio recorder using a boom-mounted hypercardioid microphone (Audio-Technica D1000HE) held approximately 20 cm in front and 45° horizontal to the mouth (where 0° is straight ahead in the horizontal plane). Sampling rate was 44.1 kHz with 16-bit quantization, and recordings were subsequently peak-amplitude normalized. The mean duration of the stimuli was 515 ms (range 401 [for the letter *D*] to 660 [for the letter *H*]).

##### Selective attention

Stimuli consisted of pure tones with a frequency of either 440 or 520 Hz, and a duration of either 50, 100, or 150 ms. The 440 Hz, 100 ms stimulus served as the *standard*, with the 50 and 150 ms tones being *short* and *long*, respectively, and the 520 Hz tones being *high*. Stimuli were generated using Praat 6.0.13 and were sinusoidally ramped over the first/last 10 ms of the onset/offset of the tone to eliminate acoustic transients.

##### Noise sensitivity

We used the Noise-Sensitivity-Questionnaire (NoiSeQ; Schutte et al., 2007) as a measure of trait noise sensitivity. The NoiSeQ is a 35-item, self-assessment questionnaire consisting of five seven-question subscales covering sensitivity to noise at home, during leisure, in communication, at work, and during sleep. Items consist of a statement such as “When people around me are noisy, I have trouble getting my work done” that are rated on a 5-point scale (*strongly agree* = 5, *agree* = 4, *neutral* = 3, *disagree* = 2, *strongly disagree* = 1). The different subscales are, at least in principle, potentially orthogonal.

##### Vocabulary

Receptive vocabulary was quantified using the Peabody Picture Vocabulary Test (PPVT-4; Dunn & Dunn, 2007). This assessment is a progressive test in which listeners select pictures from a four-picture array in response to words spoken by the experimenter. Norms exist for ages 2.6 (2 years, 6 months) to 90, but to compare across a wide range of ages, we used the raw scores rather than age-normalized standard scores.

##### Hearing

Each participant completed a standard pure-tone audiogram for both ears, administered with a GSI 18 travelling screening audiometer. Pure-tone thresholds were obtained at 250, 500, 1000, 2000, 3000, 4000, 6000, and 8000 Hz. Because of a previously unidentified intermittent noise from a thermostat in the sound booth, some participants’ right ear measures at 6000 Hz may have been unpredictably masked to at least some degree, rendering this frequency measurement unreliable. For the purposes of simplifying quantification, hearing status for each ear was computed as the mean of thresholds at 500, 1000, and 2000 Hz (pure-tone average, or PTA), and also high-frequency PTA (HFPTA) as the mean of thresholds at 2000, 4000, and 8000 Hz. The values of PTA and HFPTA for each ear were compared, and the best (lowest) value was used for each measure (i.e., we used the better PTA and HFPTA regardless of ear).

##### Personality

For purposes of comparison with previous studies, Big Five personality traits (John & Srivastava, 1999) were quantified using the BFI-10 (Rammstedt & John, 2007). Like the NoiSeQ, the BFI-10 is a self-assessment questionnaire consisting of questions such as, “I am someone who is outgoing and sociable,” to which participants respond using a 5-point scale (*strongly agree* = 5, *agree* = 4*, neutral* = 3, *disagree* = 2, *strongly disagree* = 1). Scores are averaged over the two questions associated with each subscale: Extraversion, Agreeableness, Conscientiousness, Neuroticism, and Openness.

#### Calibration task

Stimuli for establishing word recognition scores consisted of 120 semantically anomalous phrases of three to five English words (e.g., “resting older earring”; “forget the joke below”; “may the same pursued it”) with one of two different lexical stress patterns where strong (S) and weak (W) syllables alternated: SWSWSW or WSWSWS (Liss et al., 1998). They were produced by two female speakers, one a 21-year-old native speaker of English from Indiana, the other a 32-year-old native speaker of Mandarin Chinese who had been living in the U.S. for 2 years and began studying English at the age of 16 years. These 120 phrases were divided into four different (unequal) subsets. The first set, consisting of 20 phrases, was used to establish a baseline nonnative accented speech recognition score for each participant. Two additional subsets of 20 phrases each were constructed such that they were matched for number of words (82) and proportion of key words recognized correctly (69%) when produced by the nonnative accented speaker and transcribed by a panel of native listeners not otherwise involved in the study. These two sets were used in the performance verification phase of the study. The remaining 60 phrases were used in a staircase task to establish an SNR level likely to support equivalent word recognition scores when listening to native-accented speech in noise.

#### Story comprehension

Stimuli for the comprehension task consisted of 12 short stories taken from the Discourse Comprehension test (Fossett et al., 2004). Each story is accompanied by a set of 10 multiple-choice questions about their content. The stories were recorded by the same two talkers who produced the phrases used in the word recognition task. The 12 stories, as produced by the nonnative accented talker and unmasked by noise, were presented to a panel of listeners not otherwise involved in this research, who then answered the test questions. On the basis of these results, the stories were grouped into two sets of five stories with roughly equal mean performance across the two sets, and two additional practice stories.^1^ Using the two sets, it was then possible to counterbalance story sets across conditions, so that every story was heard approximately equally often in the noise and accented conditions across listeners, but there should be no a priori difference in expected baseline performance.

### General procedures

Participants were asked to get a good night’s sleep, to not consume alcohol the night before the study, to not consume an unusual amount of caffeine (either more or less than usual) on the day of the study, and to refrain from consuming any caffeine within 2 hours prior to the physiological measurement session. Testing times ranged from about 9 a.m. until about 6 p.m., but due to constraints on lab, researcher, and participant availability, it was not possible to ensure that participants always completed both sessions at the same time of day. However, as physiological measures were collected only during the second session, circadian variation in autonomic responsiveness should add variability only between and not within subjects.

Participants completed the study in two sessions, spaced at least one day apart. In the first session, participants completed all of the background questionnaires and testing to establish individual differences variables. They also performed the word recognition tasks designed to estimate SNR levels needed for the comprehension task. In the second session, participants were first reacquainted with the methods and goals of the study, and then most participants also took the offered opportunity to void their bladder (no records were kept) in an attempt to reduce variability in physiological responsiveness due to hydration and/or discomfort while sitting still for a long time. Sensors were attached, and the *story comprehension* task was conducted. The two sessions were each completed within 2 ½ hours (≤5 hours total participation). Following the comprehension task, participants were debriefed and paid for their participation. All activities in this experiment were conducted under a protocol approved by the human research subject protection program at Purdue University.

#### Working memory capacity task

In an *N*-back task, stimuli are presented at a regular rate, and participants are asked to respond when a given stimulus matches a specific target condition. In the 0-back condition, the target is a designated stimulus (e.g., the letter *Q*). In the 1-back condition, a target is any stimulus that matches the stimulus immediately before it (i.e., one stimulus back in time), and in the 2-back condition, targets are those stimuli identical to stimuli two steps back in time.

Participants completed five blocks of trials. The first block was 0-back, the second and third were 1-back, and the last two were 2-back blocks. The entire test took less than 15 minutes. There were 50 stimuli per block. Forty of these were nontargets, and 10 were targets. Targets were interspersed randomly within the sequence, except that they could not appear in the first or last position in the block (nor in the first two positions for the 2-back blocks). Stimuli were presented at a uniform rate with a 1,000 ms interstimulus interval (ISI). Responses made fewer than 100 ms or more than 1,500 ms after the onset of the stimulus were treated as nonresponses (i.e., counted as misses for target stimuli, or as correct rejections for nontargets). Participants responded by pressing a button using their dominant index finger on a response box (Cedrus RB-730) for each target. Stimuli were presented diotically at a comfortable listening level (approximately 65 dB SPL) via Sennheiser HD 380 Pro headphones using a script written for Presentation 18.2 Build 01.04.16.

#### Selective attention task

In this task modeled on experiments first presented by Dalton and Lavie (2004), listeners heard a series of tone pips, one of which (the target) was always a different duration than the others (the standards). In some trials, one of the nontarget stimuli was produced at a different pitch (a distractor). The rationale of the task was that, in trials with distractors, listeners should be slower to respond correctly to the target because selective attention is drawn toward the irrelevant pitch dimension by the distractor. Thus, individual differences in the degree of slowing in distractor-present versus distractor-absent trials serve as a measure of individual susceptibility to auditory distraction.

On each trial, seven stimuli were presented with equal onset intervals (185 ms). On each trial, either the third or fourth stimulus was a different length than the other six tones in the trial. The standard stimuli were always 440 Hz in frequency, and 100 ms in duration, while the different stimulus was always either *shorter* (50 ms) or *longer* (150 ms) than the standard. Participants were asked to press a button on a response box (Cedrus RB-730) using their thumbs (one held on each button) indicating whether the stimulus that was a different length was shorter (left button) or longer (right button). One third of all trials (32) were *no-distractor* trials in which all the other stimuli were standards (440 Hz, 100 ms). One third of all trials were *target* trials, in which the target stimulus was a high tone (520 Hz). One third of all trials were *distractor* trials, in which the target was at the standard frequency (440 Hz), but there was a standard-length (100 Hz) high tone (520 Hz) presented either immediately preceding or immediately following the target. Stimuli were presented diotically via Sennheiser HD 380 Pro headphones using a script written for Presentation 18.2 Build 01.04.16. Levels were the same as for the working memory task (above) though were not measured here due to the very short nature of the stimuli. Participants completed four blocks of 24 trials each (96 trials total, 32 *no-distractor* trials, 32 *distractor* trials, and 32 *target* trials).

#### Estimating noise levels for equivalent word recognition performance

In studies of listening effort across different conditions, it is typically considered important to ensure that participants are performing equivalently in all conditions so that self-assessment of performance does not confound measures of effort (Picou, Bean, Marcrum, Hornsby, & Ricketts, 2019). In an attempt to approximately match performance across the two tasks for each individual, we used a multistage approach to (1) assess recognition of words produced by the nonnative speaker; (2) estimate two signal-to-noise ratios for achieving word recognition scores for native-accented speech presented in speech-shaped noise that were toward the upper and lower end of the individual’s abilities; (3) using linear interpolation between these two points, estimate an SNR for the native-accented speech that would result in similar performance compared with nonnative accented speech.

Phrases were presented to listeners via a single loudspeaker (Hafler M5 reference) placed about 1.5 m directly ahead at head height while they were seated in a comfortable chair in a single-walled sound booth (IAC Inc.). Ambient level in the booth was below 30 dBA measured at a point centered over the chair where participants were seated, at approximately head height (directly in line with the stimulus speaker). Sound levels for the unmasked nonnative accented talker and for the combined native accented talker plus masking noise stimuli were maintained at roughly 66 dBA measured at the same location. Trial timing was dependent upon participants’ speed of response, in that the experimenter pressed a key to start each trial after the participant had finished the previous trial. During the task, participants watched a computer screen at approximately 2-m distance displaying a grey background with a centered, 64-pt Tahoma font “+” symbol (subtending approximately 0.5° of visual angle). They were instructed to sit still and watch for the cross to change to an asterisk (“*”) to signal when it is time to speak.

##### Establishing nonnative accented speech performance

Participants first heard and repeated 20 phrases produced by the nonnative accented talker that were presented in quiet. Each trial began with a 250-ms silent pause before the start of the phrase to ensure that there was sufficient time between trials. After the speech ended, there was a 500-ms pause, after which the fixation cross on the computer screen changed to an asterisk, indicating the participant should respond. Each response was scored online by the experimenter in terms of number of correct words. Words had to match the target exactly to be counted as correct (i.e., words with added or deleted morphemes were counted as incorrect). Following the registration of the number of correct words, there was a 1.5-s pause, the asterisk changed back to a cross, and the next trial began. Because entering the number of correctly repeated words took a variable amount of time, the intertrial interval varied. However, it was never less than 1.75 s.

##### Estimating noise-masking levels for lower and higher performance

To determine a likely SNR at which an individual participant’s performance for the noise condition would be matched to their performance in the accented one, an interleaved staircase method of adjustment was used (Levitt, 1971). Participants performed the same phrase recognition task as described in the previous section with a few key differences. First, the target phrases were produced by the native speaker and were presented in a speech-shaped noise masker that began between 3.5 and 4.5 seconds before the phrase began to allow listeners time to adapt to the presence of the noise. Duration of the noise before speech onset was jittered between 3.5, 4.0, and 4.5 s across trials, with a mean value for the entire block of 4 s. The noise also continued 500 ms longer than the speech, at which point the fixation cross changed to an asterisk, indicating that participants should respond.

**Fig. 1:**
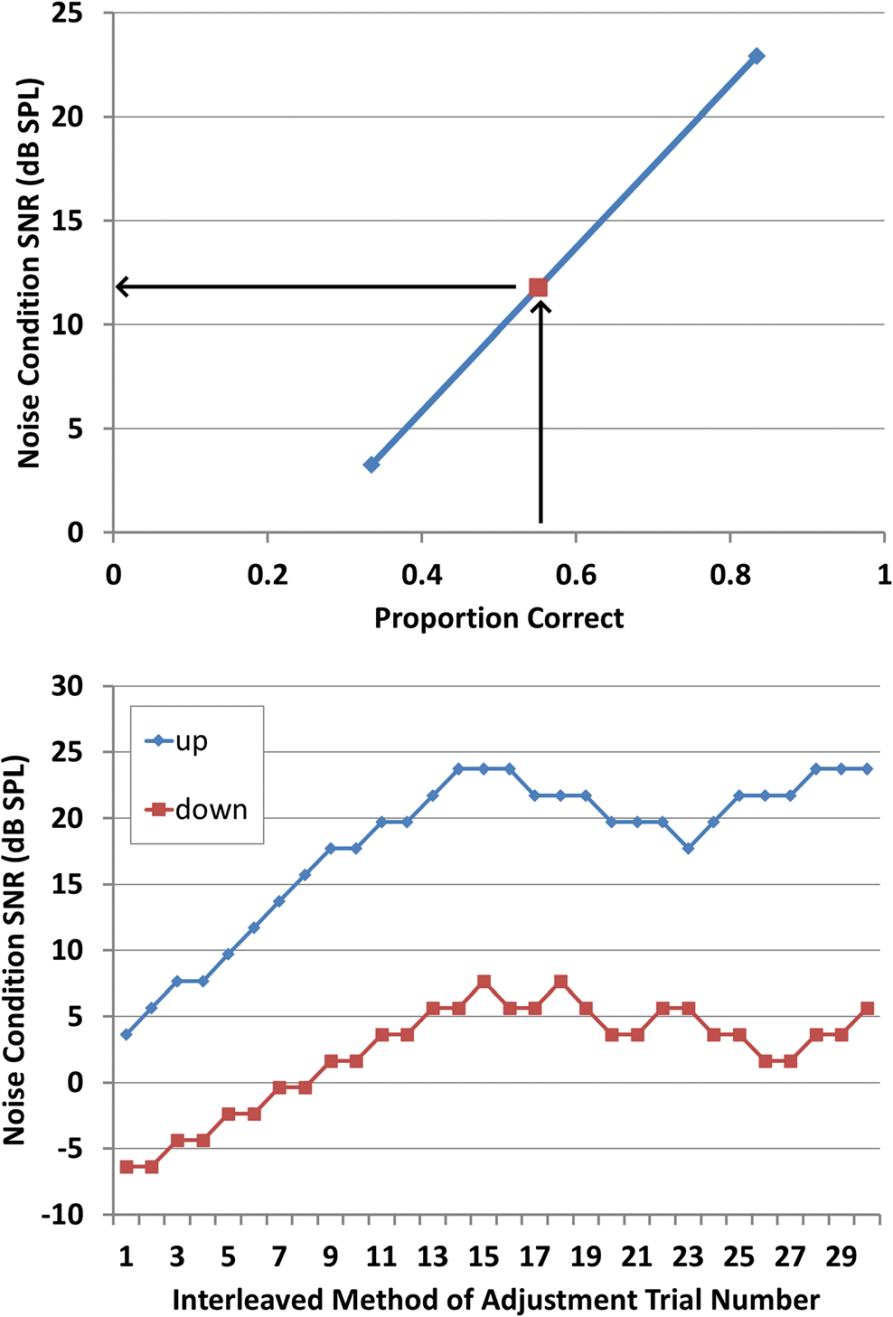
Example of identifying lower and upper bounds for performance on the noise-masked, native-accented phrases. Top panel: Final signal-to-noise ratio (SNR), averaged over last five trials of each run, for the low-performance and high-performance runs, with interpolated value (red dot) for matching performance on nonnative accented speech. Black arrows illustrate conceptual process for relating measured proportion correct words recognized in the accent condition to interpolated SNR value for use in the noise condition. Bottom panel: SNR in the two interleaved runs of staircase trials at each trial (*x-*axis). (Color figure online)

Second, participants completed a total of 60 trials in this task, consisting of two interleaved, 30-trial “staircases” following the methods of Levitt (1971).^2^ Over the course of the 30 trials in each staircase, the SNR on each trial was adjusted depending on participants’ accuracy on preceding trials in the staircase. The two staircases were designed to approach 79.4% correct (using a “3 up, 1 down” adjustment scheme) and 29.3% correct (using a “1 up, 2 down” scheme), respectively. SNR was adjusted by changing both the signal and noise level to maintain a similar overall signal intensity, as some physiological measures are sensitive to overall level. Once the 60 trials were completed, the mean signal and noise levels as well as mean proportion of words repeated correctly on the last five trials of each staircase were computed. Using these measures, a linear function was computed connecting the two experimentally established points (SNR-by-proportion correct), and this function was used to determine an SNR likely to exhibit performance similar to that of the accented condition (see Fig. 1). Note that, although this method could conceivably result in extremely high or low SNR levels for extreme levels of performance in the accented condition, minimum target level and maximum noise level were capped at 55.3 and 61.7 dB SPL, respectively, permitting a minimum SNR of −6.4 dB SPL.^3^

##### Verification of word recognition in the two conditions

To verify that performance on the two conditions was roughly matched, two sets of 20 phrases were presented to participants using a method similar to that of the original block of 20 trials with nonnative accented speech. The SNR for the 20 noise-masked trials was determined by interpolating between the two SNRs established using the staircase procedure and the target level in both conditions was set at that determined by the same procedure. In addition, for both blocks of trials there was a 12-s silent pause following the end of each phrase, and only after this pause did the fixation cross change to an asterisk indicating that participants were to respond. The original purpose of this pause was to permit any autonomic systems responding to the challenge of the listening task to recover. However, due to changes in experiment protocol (shifting this assessment to the first test session), no autonomic responses were recorded. As before, participant responses were scored by an experimenter seated outside the booth listening through headphones connected to a microphone mounted on the ceiling of the booth above the participant.

#### Story comprehension

Story comprehension was the experimental task during which physiological and self-report measures of listening effort and comprehension were assessed. Participants heard short stories in two conditions, noise-masked and nonnative accented, and then answered questions about them.

After entering the sound booth, participants were seated in a comfortable chair and leads for recording physiological data were attached to the Biopac MP150 system. A short calibration test was run to ensure that all signals were being acquired at adequate levels. The lights were dimmed (approximately 18.6 lux with computer screen off), and participants watched a short (3 min) portion of a relaxation video (Isis Visuals, Inc.) showing waves gently rolling onto a beach. The task was presented in two blocks, one for each condition. Order of blocks was counterbalanced across participants, as was the set of stories used in each block. Each block consisted of one practice trial followed by five test trials. During each trial, participants first heard a story, and then answered 10 multiple-choice questions about it. Questions were presented via text on a 50-in. computer screen suspended on the wall of the booth approximately 2 m directly in front and slightly above head height of the participant. In the noise condition, the noise began 3 seconds before the speech, ended at the same time as the speech, and was not present during the test questions. Questions were presented one at a time, visually on the computer screen. In the practice trials, participants received visual feedback and the correct answer on each question. During the test trials, there was no feedback. Responses were made via button press (Cedrus RB-730) using only the dominant hand.

After each block, participants also filled out a short questionnaire (NASA TLX; Hart & Staveland, 1988) about their perceived task load during the preceding listening task. Responses were made on a 20-point equal-appearing interval scale to the question on effort (“How hard did you have to work to accomplish your level of performance?”) and were used as a subjective measure of listening effort for each block.

### Physiological data

Physiological data were recorded only during the story comprehension task using a Biopac MP150 system with associated modules. All modules included a comb filter with 50 dB rejection @ 60 Hz (and harmonics up to the Nyquist limit) to remove any noise related to the use of mains power. All electrical connections between mains-powered equipment and the research participant were isolated using optical couplings (Biopac INISOA).

#### Electrodermal activity (EDA)

EDA was recorded using a GSR100C module with gain set to 10 μS/V and an initial sampling rate of 1.25 kHz. Skin on the thenar and hypothenar eminences of the nondominant hand was first cleaned with distilled water prior to placing two 11mm pregelled isotonic Ag/AgCl electrodes (BIOPAC EL507). Additional BIOPAC Gel101 was added to the electrode sponge pad at time of application as needed, per BIOPAC recommendations.

#### Heart period

Heart period was calculated from the Electrocardiogram (ECG) recorded using an ECG100C module with input voltage range set to ±2 mV (gain setting of 5,000) at a sampling rate of 1.25 kHz, using a Lead II configuration (positive electrode on the right forearm, negative on the left calf, and ground on the left forearm). Skin on the left and right forearm and left calf near the ankle was scrubbed with a commercial alcohol prep pad containing 70% isopropyl alcohol. Scrubbing was vigorous but did not damage the skin. Exact electrode sites were chosen to avoid as much as possible areas with hair, moles, tattoos, acne, etc. Electrodes were 11 mm disposable, pregelled (7% chloride salt) general-purpose Ag/AgCl electrodes (BIOPAC EL503). Additional BIOPAC GEL100 gel (5%, 0.85 molar NaCl) was added as needed to fully saturate the sponge.

#### Peripheral vasoconstriction

The amplitude of the blood volume pulse (BVPA) was computed from a photo pulse plethysmographic signal via a Biopac PPG100C module with a gain set to 100, a low-pass filter cutoff of 10 Hz, and a high-pass filter cutoff of 0.5 Hz. A reflective-method transducer (Biopac TSD200 or TSD200C) consisting of side-by-side infrared emitter, and photodiode detector was affixed the palmar side of the distal phalange of the index finger of the nondominant hand using a Velcro strap.

#### Facial electromyography (EMG)

Here, we measured activity in corrugator supercilii via a Biopac EMG100C module with input voltage range set to ±2 mV (gain setting of 5,000). Skin superior to the interior corner of the left eye was gently scrubbed with a commercial alcohol prep pad containing 70% isopropyl alcohol. Electrode sites were located immediately above the inner corner of the left eye, and then approximately 1 cm laterally and slightly superior to this location (Fridlund & Cacioppo, 1986), taking care in both cases to minimize the amount of adhesive contacting hair of the eyebrow. Electrodes were 4 mm reusable Ag–AgCl shielded electrodes (Biopac EL254s) filled with conductive gel (5%, 0.85 molar NaCl) and affixed with a small adhesive ring.

### Analyses

#### Working memory capacity task

Performance was quantified as the sensitivity index *d'* using the formula: *d'* = *z*(Hits) − *z*(False Alarms; Macmillan & Creelman, 2004), where Hits refers to the proportion of target trials on which the participant responded correctly, and False Alarms refer to the proportion of nontarget trials on which the participant failed to withhold their response (i.e., responded incorrectly). Perfect scores were adjusted using the substitution of 1 − 1/(2*n*) for Hits = (1.00), and 1/(2*n*) for False Alarms = (0.00), where *n* is the total number of trials (Macmillan & Creelman, 2004). Working memory capacity was calculated as the average *d'* score across the two 2-back blocks because participants were often at ceiling in the 0-back and 1-back blocks.

#### Selective attention task

As a measure of distractibility, we calculated the difference in response time between correct responses on no-distractor trials and distractor trials, such that a larger positive number indicates greater susceptibility to distraction (i.e., longer RT on *distractor* trials than on *no-distractor* ones).

#### Data reduction and preprocessing of individual differences

For subsequent data analysis, principal components analysis (PCA) was used to reduce the complexity of the individual differences variables for those categories of variable assessed using multiple measures (see Table 3), following the methods employed by Humes, Busey, Craig, and Kewley-Port (2013). These multicomponent variables consisted of five *personality* variables (the Big Five variables of openness, conscientiousness, extraversion, agreeableness, and neuroticism; John & Srivastava, 1999), four *noise sensitivity* variables (the NoiSeQ subscales of Work, Communication, Sleep, Home Environment, and Leisure; Schutte et al., 2007), and two *hearing* variables (PTA and HFPTA in the best ear). PCA results (see below) suggested the use of three components as representative of personality, and one each for noise sensitivity and hearing acuity.

Although as shown in Table 3, there were three sound level*-*related variables for each participant (SNR, background noise level, and target level [used in both conditions]), these were perfectly correlated because both target and noise level were adjusted simultaneously to determine SNR. Therefore, SNR was used as the sole level-related measure. In addition, individual scores were included for *age*, *working memory capacity*, *selective attention*, and *vocabulary*. All of these single-measure variables were scaled and centered prior to inclusion in multilevel modeling analyses (below) to permit them to range on a scale more comparable to that of the PCA component dimensions derived from the other variables. Centering and scaling can also help with model convergence and comparability of coefficients across predictors in multilevel modeling (below; Bates, Mächler, Bolker, & Walker, 2015; Eager & Roy, 2017).

#### Physiological data

For all physiological values, log ratio scores were used. To compute these scores, the raw measurements as described below were normalized against the same values calculated during two baseline periods, one conducted before and one after the two blocks of stories. Each baseline period consisted of the middle 3 minutes of the same 5-minute relaxation video. This normalization resulted in a ratio indicating change from baseline, with values >1 indicating an increase in the measure compared with baseline, and values <1 representing a decrease. The logarithm (base 10) of this ratio was then used as the input in all statistical analyses.

##### Electrodermal activity

Following data collection, the EDA signal was smoothed and down-sampled to 25 Hz. To identify SCRs, the raw SCL signal was detrended using a 0.05 Hz high-pass filter. SCRs were identified as peaks in the detrended signal having an amplitude >0.03 μS and also having an amplitude in the raw SCL recoding at least equal to 10% of the peak amplitude observed across the entire block. Raw measures of EDA consisted of overall skin conductance level (SCL) averaged over the listening portion of the trial, and rate of spontaneous SCR events per second across the trial. This resulted in two physiologically related, electrodermal measures, SCL and SCR rate, that we interpreted as measures of (sympathetic) arousal.

##### Heart period

Following data collection, the signal was down-sampled to 625 Hz. R-wave peaks were marked automatically using a script written in AcqKnowledge 4.4.1, and any errors in placement were corrected by hand. Following recommendations of Velden and Wölk (1987), interpeak interval duration was stored as a wave such that the value, in milliseconds, of each cycle was represented for the entire duration of the cycle. Average heart period was then calculated from this representation and interpreted as an indicator of arousal.

##### Peripheral vasoconstriction

The PPG100C signal was sampled at 1.25 kHz and subsequently smoothed (200 point/50 ms window, mean value). Peak-to-trough amplitude was computed for each period using the default cycle detector in AcqKnowledge 4.0. Peaks lower than 0.1 V were ignored, and obvious errors were corrected by hand following visual inspection of the output. Correction consisted of linear interpolation across misidentified peaks, peaks based on movement artefacts, and other signal irregularities. The average of all peak-to-trough amplitudes (mean BVPA) in a given listening period was interpreted as a measure of sympathetic arousal.

##### Facial electromyography

The EMG signal was sampled at 1.25 kHz, then bandpass filtered between 20 and 600 Hz using a Hamming window (van Boxtel, 2001) with optimal coefficients (as determined by default AcqKnowledge 4.4 settings). This signal was rectified by taking the absolute value, and then smoothed by taking the median of a 10 ms (125 sample) sliding window. Finally, following visual inspection of the output obvious errors were removed and the excised regions were linearly interpolated. The mean value of this rectified signal over a given listening period was used as an indicator of valence.

#### Modeling analyses

To identify relationships between relevant predictor variables and measures of performance and listening effort, we employed multilevel mixed effects modeling (*lmer*) implemented in *nlm4* (Version 1.1-17; Bates et al., 2015) within the R (Version 3.5.1) programming environment (R Development Core Team, 2018). The fitted model was subsequently submitted to analysis of deviance testing using Type II Wald *F* tests with Kenward-Roger degrees of freedom via the *car* package (Version 3.0.0; Fox & Weisberg, 2019). For all dependent measures except TLX scores of effort (that were collected only once per block), both story and participant were included within the random effects structure (see below).

## Results

### Individual differences

Results showed relatively wide ranges of individual differences across most variables (see Table 2).

**Table 2:** Descriptive statistics for raw, unscaled individual differences measures

Measure	Min	Max	Mean	*SD*	Median	IQR
**Age**	19	68	35.63	16.75	27	24.75
**WMC(***N*** = 33)**	0.97	3.29	2.16	20.61	2.12	1.00
Attention	−294.75	485.88	93.23	176.90	70.97	181.05
**Vocabulary**	191.0	225.0	213.6	7.91	214.0	10
**Personality**						
Openness	5	10	7.23	1.37	8.0	2.0
Conscientiousness	5	10	8.43	1.17	8.0	1.0
Extraversion	3	10	6.97	1.71	7.0	2.0
Agreeableness	5	10	7.60	1.31	8.0	1.0
Neuroticism	2	9	5.0	1.83	5.0	2.0
**Noise sensitivity**						
Work	1.57	5.00	2.74	0.75	2.71	1.0
Communication	1.57	4.29	2.60	0.59	2.43	0.71
Home	1.00	4.14	2.73	0.73	2.71	0.57
Leisure	1.57	3.71	2.71	0.52	2.71	0.57
Sleep	1.14	4.86	2.61	0.89	2.57	1.14
**Hearing**						
PTA	−8.33	10.00	0.58	4.56	0.00	8.33
HFPTA	−10.00	28.33	−1.33	9.67	−3.33	12.50
**Sound levels**						
Target Leq	53.31	59.55	56.85	1.53	56.93	2.62
Noise Leq	57.50	63.70	60.19	1.52	60.1	2.61
SNR	−10.39	2.0	−2.05	3.34	−3.17	5.23

*Note.* Age in years; IQR = interquartile range; WMC = *d'* score on the 2-back task; Attention = ms difference between distractor-present and distractor-absent trials in the Dalton and Lavie (2004) task; Vocabulary = PPVT-R raw score; Personality variables refer to Big Five measures; Noise Sensitivity subtest scores from the NoiSeQ; PTA = better-ear pure-tone thresholds averaged over 500, 1000, and 2000 Hz tones; HFPTA = better-ear pure-tone thresholds averaged over 2000, 4000, and 8000 Hz; Target Leq and Noise Leq are included here only for reference. All calculations involving level were computed using SNR values alone

#### Reducing parameters (principal components analysis)

To reduce the number of parameters to be included in the multilevel modeling analysis, each group of predictor variables (personality, noise sensitivity, hearing) was submitted to principal components analysis using the FactoMineR package (Version 1.34; Lê, Josse, & Husson, 2008) implemented within the R (Version 3.5.1) programming environment (R Development Core Team, 2018). Only those principal components with eigenvalues greater than 1 were retained for further analysis (see Table 3). A summary of the results of the PCA analyses relevant to determining the number of dimensions included in further analyses is provided in Table 3.

**Table 3:** Results of reducing predictor dimensions via principal components analysis

Parameter group	Initial no. of variables	Number of dimensions with eigenvalues > 1.0	Cumulative percentage variance accounted for by these variables	Contribution of original factors to each dimension (in %) (>10%)	Loadings
Personality Openness Conscientiousness Extraversion Agreeableness Neuroticism	5	3	77.42	D1 (1.43) E: 46.5% A: 38.6% C: 11.0% D2 (1.27) O: 57.8% C: 31.6% D3 (1.17) N: 61.5% C: 20.3%	D1 E: 0.682 A: 0.621 C: 0.331 D2 O: 0.760 C: −0.562 D3 N: 0.784 C: 0.451
Noise Sensitivity Work Home Leisure Communication Sleep	5	1	59.75	D1 (2.99) Leisure: 23.7% Work: 23.2% Comm.: 19.8% Home: 18.3% Sleep: 15.1%	D1 Leisure: 0.486 Work: 0.482 Comm.: 0.445 Home: 0.427 Sleep: 0.388
Hearing Best PTA Best HFPTA	2	1	79.41	D1 (1.59) PTA: 50% HFPTA: 50%	D1 PTA: 0.891 HFPTA: 0.891

This analysis reduced the total number of predictor variables from 18 to 10. The combined predictor variables derived from PCA were Personality Dimension 1 (related to extraversion, agreeableness, and neuroticism traits), Personality Dimension 2 (related to openness and conscientiousness traits), Personality Dimension 3 (related to neuroticism, conscientiousness, and openness), Noise sensitivity, hearing (related to thresholds, thus higher values reflect poorer hearing), and SNR (which was also directly related to the target and noise levels in the noise condition, and to the target level in the accented condition). There were an additional four predictors each based on a single measurement or test consisting of age, attention score, working memory score, and vocabulary score.

### Correlations between performance, effort, and physiological responses

**Fig. 2:**
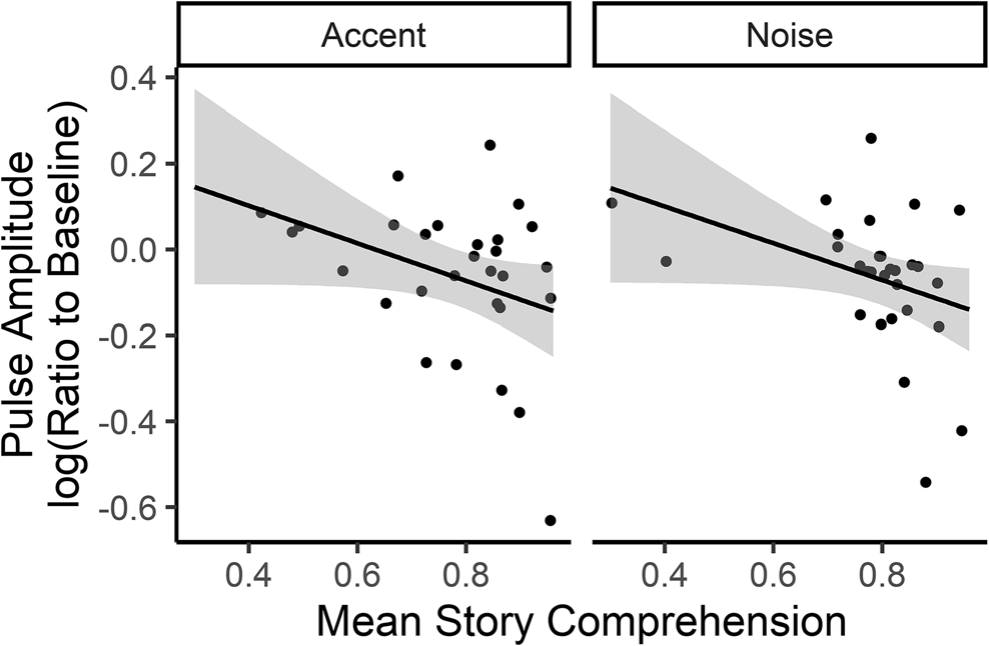
Relationship between mean performance score (per block) and mean log of the ratio of blood volume pulse amplitude (BVPA) during the task to baseline BVPA, displayed by condition

One concern in the study of listening effort is that various measures may reflect something other than, or in addition to, the engagement of cognitive resources, especially internal assessments of performance (Picou et al., 2019) or affective response to task conditions or demands (Francis and Love, 2020). In addition, recent research demonstrates that self-report and physiological measures of listening effort may not, in fact, be reflecting the same underlying processes, or at least not doing so to the same degree (Alhanbali et al., 2019; Lau et al., 2019; Strand et al., 2018). To examine these possibilities in the present data, we compared Pearson product-moment correlations between performance scores (proportion correct on the story-related questions), self-rated effort (NASA TLX Effort subscale scores) and normalized changes in physiological measures related to effort (see Table 4) using the *cor.test* function. Results showed that the only significant correlation was that between normalized blood volume pulse amplitude (BVPA; a measure of sympathetic arousal) and performance, such that individuals who showed a larger decrease in BVPA from baseline period to the listening task also achieved higher scores on the comprehension tests. However, as shown in Fig. 2, this correlation was comparable across both conditions. These findings suggest a relationship between tonic sympathetic arousal and performance, which can be evaluated in further detail by examining the relationship between other sympathetically controlled physiological responses.

**Table 4:** Correlations between performance measures (accuracy scores on story comprehension questions) and self-rated effort scores (NASA TLX Effort subscale), as well as correlations of both performance measures and self-rated effort scores with physiological measures associated with listening effort

		*r*	*t*	*df*	*p*
Performance score	Effort	−0.096	0.80	68	.429
	SCL	0.062	0.52	68	.608
	SCR rate	0.043	0.32	54	.751
	RR interval	0.001	0.01	68	.992
	**BVPA**	**−0.357**	**2.86**	**56**	**.006**
	Corrugator	−0.057	−0.47	68	.640
Rated effort	SCL	−0.030	0.25	68	.803
	SCR rate	0.004	0.03	54	.973
	RR interval	−0.148	1.24	68	.220
	PPG	0.107	0.81	56	.423
	EMG	−0.107	−0.89	68	.377

*Note*. SCL = skin conductance level; SCR rate = rate of spontaneous skin conductance responses per second; RR interval = period of the heart rate assessed via electrocardiogram (Lead II); BVPA = blood volume pulse amplitude, assessed with finger-tip pulse plethysmography; Corrugator = mean rectified amplitude of activity in corrugator assessed with facial electromyography. The correlation marked in boldface is significant at the *p* < .05 level

In this data set, in addition to BVPA, we collected two other measures associated with listening effort that are exclusively controlled by the sympathetic nervous system: SCL and SCR rate. While task-related changes in these two EDA measures were strongly correlated, there was no correlation between measures across sympathetic end organs (BVPA, reflecting peripheral vasoconstriction vs. the two EDA measures reflecting eccrine sweat-gland activity), as shown in Table 5.

**Table 5:** Correlations between exclusively sympathetic measures related to listening effort

		*r*	*t*	*df*	*p*
SCL	SCR rate	0.30	4.14	195	<.001
SCL	BVPA	−0.052	0.88	286	.378
SCR rate	BVPA	0.045	0.55	149	.580

*Note*. SCL = skin conductance level; SCR rate = rate of spontaneous skin conductance responses per second; BVPA = blood volume pulse amplitude, assessed with finger-tip pulse plethysmography; Corrugator = mean rectified amplitude of activity in corrugator assessed with facial electromyography. Correlations marked in boldface are significant at the *p* < .05 level

### Relationships between predictors, performance, and effort

To evaluate the relationships between relevant predictor variables and measures of performance and listening effort, mixed effects models were fitted, with results described according to each dependent measure. The overall structure of each model (in *lme4* syntax) was as follows, where “Score” refers to the dependent measure of interest and “Condition” refers to the source of the listening challenge (i.e., nonnative accented speech or noise masking). The factors containing the term “dim” refer to variables derived from principal components analysis of the respective individual differences variables (i.e., “personality_dim1” refers to the first principal component derived from the Big Five personality trait assessment). Note the “*” operator includes interactions and lower-order effects, meaning that the *lme4* package automatically calculates all simple effects contained within these terms, so simple effects for Condition and for each of the individual difference variables were calculated as well. Missing variables (i.e., the two participants with missing WMC data) were handled by pairwise deletion according to default *lme4* procedures.

(1) trialLevel.model = lmer(Score ~

personality_dim1*Condition +

personality_dim2*Condition +

personality_dim3*Condition +

noiseSensitivity_dim*Condition +

hearing_dim*Condition +

scaled_SNR*Condition +

scaled_Age*Condition +

scaled_Attention*Condition +

scaled_WM*Condition +

scaled_PPVT*Condition +

(1 + Condition|Subject) +

(1 + Condition|Story),

data = dataset, REML=TRUE)

Anova(trialLevel.model, test-statistic = "F")

For the TLX score of rated effort that were collected for each block, the model was as follows. Note that the two models differ only with respect to the random effects terms, necessary because TLX scores were collected only at the block trial and not trial level.

(2) blockLevel.model = lmer(scaled_Effort ~

personality_dim1*Condition +

personality_dim2*Condition +

personality_dim3*Condition +

noiseSensitivity_dim*Condition +

hearing_dim*Condition +

scaled_SNR*Condition +

scaled_Age*Condition +

scaled_Attention*Condition +

scaled_WM*Condition +

scaled_PPVT*Condition +

(1 | Subject) +

(1 | Condition),

data = dataset_means, REML=TRUE)

Anova(blockLevel.model, test-statistic = "F")

Note that all *p* values associated with both models were calculated using the *car* 3.0.0 package using Type II Wald *F* tests with Kenward-Roger degrees of freedom. In all cases, in the interest of brevity, only statistically significant main effects and interactions are reported. All unreported effects are nonsignificant.

#### Mean proportion correct (story questions)

**Fig. 3:**
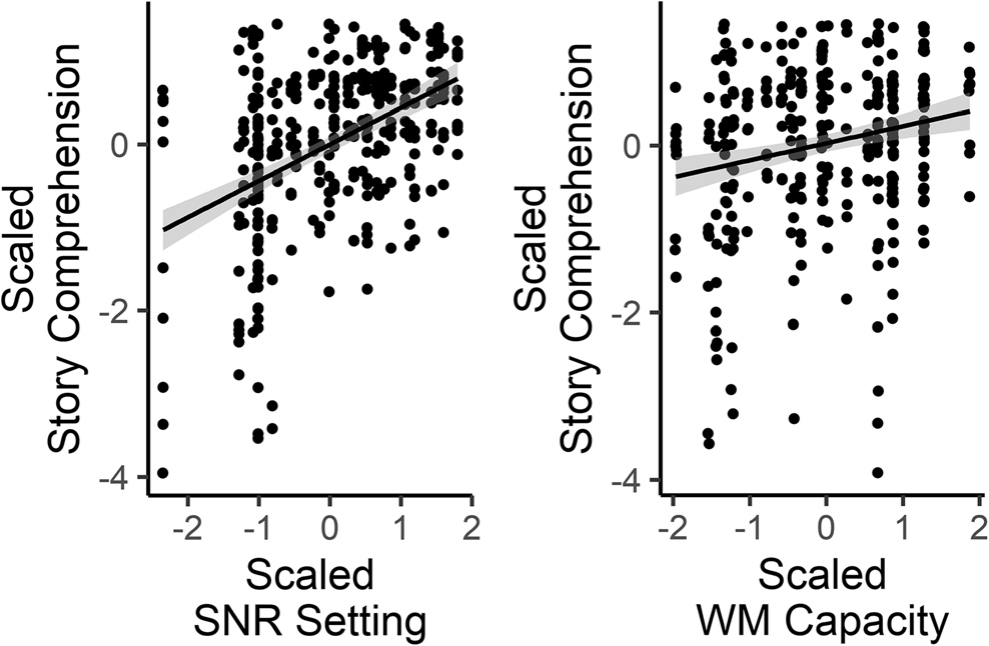
Scaled and centered story comprehension scores versus scaled and centered SNR and WM capacity scores, by subject (*N* = 35) and trial (10, across two blocks). Each dot represents a single trial by a single participant. Lines represent linear regression with shaded 95% CI. SNR = signal-to-noise ration; WM = working memory

Participants scored an average of 78.4% correct (*SD* = 13.1, range: 30%–96%) on the story comprehension questions, but did not differ appreciably between the two conditions (accent = 77.9%, *SD* = 13.4; noise = 78.9%, *SD* = 13.0). Results of the analysis of the linear model (using scaled and centered values) showed only significant contributions from SNR, *F*(1, 22.12) = 38.12, *p* < .001, and the working memory score, *F*(1, 21.85) = 10.02, *p* = .005, to performance on the story comprehension questions (see Fig. 3). These results suggest that as SNR increases across participants, so, too, does story comprehension. Similarly, as individual participants’ WM capacity increases, story comprehension scores also increase. The lack of any main effect of, or interaction with, condition suggests that listeners’ comprehension did not differ significantly as a function of the listening challenge (nonnative accent vs. noise masking), likely due to the attempt to match lexical recognition performance across the two conditions for each individual.

#### Listening effort

Overall, most self-reported and physiological measures related to listening effort showed significant contributions to their respective models. For blood volume pulse amplitude (BVPA) and facial EMG (corrugator supercilii), there were changes from baseline, but there were no interactions with Condition, nor any contributions of any other factors to the models constructed for these variables, so these measures will not be analyzed further.

#### NASA TLX Effort subscale

**Fig. 4:**
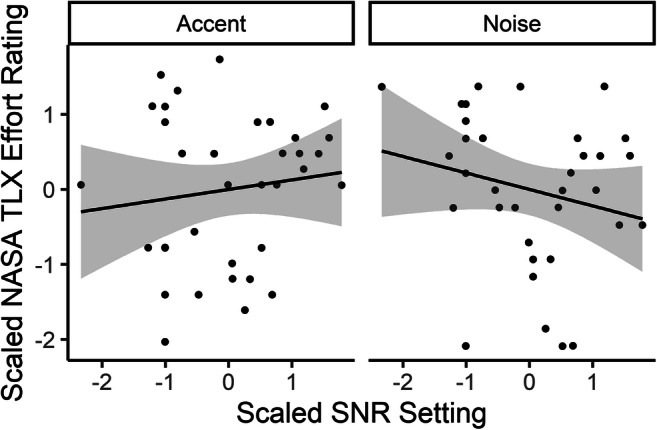
Reported effort (NASA TLX Effort subscale) by each participant’s individual signal-to-noise ration (SNR) setting (directly related to target level in the accent condition). Each symbol represents a single block for a single participant. Lines represent linear regression with shaded 95% CI

Participants showed an overall higher rating of effort in the noise condition (mean = 14.1, *SD* = 4.37, range: 5–20) than in the accented condition (mean = 11.7, *SD* = 4.82, range: 2–20). Results of the analysis of the linear model showed a significant interaction between SNR (also related to target level in the accented condition) and condition, *F*(1, 22) = 9.03, *p* = .007. This interaction is shown in Fig. 4. In the noise condition, higher SNR levels were rated as less effortful (right panel). By contrast, in the accented condition, in which the level dimension corresponds to increasing target level in the absence of noise, higher target levels were rated as more effortful.

#### Electrodermal activity

##### Skin conductance level

**Fig. 5:**
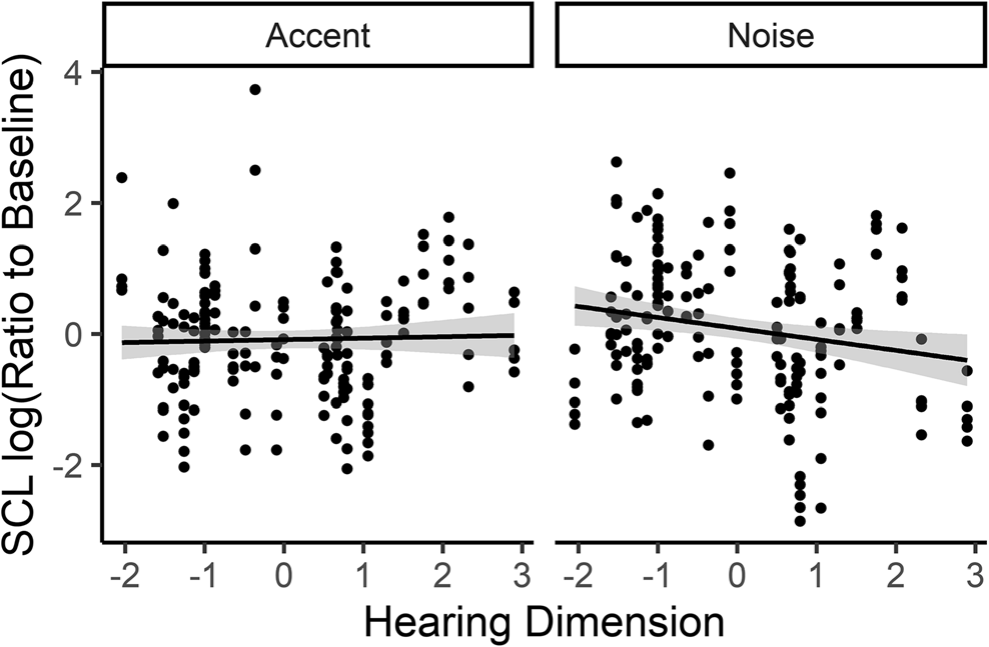
Hearing dimension versus scaled and centered log ratio of skin conductance level (SCL), by condition. Each symbol represents a single trial for a single participant. Lines represent linear regression with shaded 95% CI

Participants tended to show little change in mean skin conductance level compared with baseline, with log ratios averaging 0.023 (*SD* = 0.052, range: −0.13–0.13), and did not differ much between the two conditions (accent = 0.023, *SD =* 0.046; noise = 0.034, *SD* = 0.059). Results of the analysis of the linear model showed a significant interaction between the hearing composite dimension and condition, *F*(1, 21.96) = 5.33, *p* = .031. As shown in Fig. 5, this interaction was mainly due to a slight decrease in skin conductance level in the noise condition as the hearing dimension increased, suggesting that listeners with poorer hearing (higher thresholds) tended to be less aroused in the noise condition, but this relationship did not hold in the accented condition.

##### Rate of skin conductance response

**Fig. 6:**
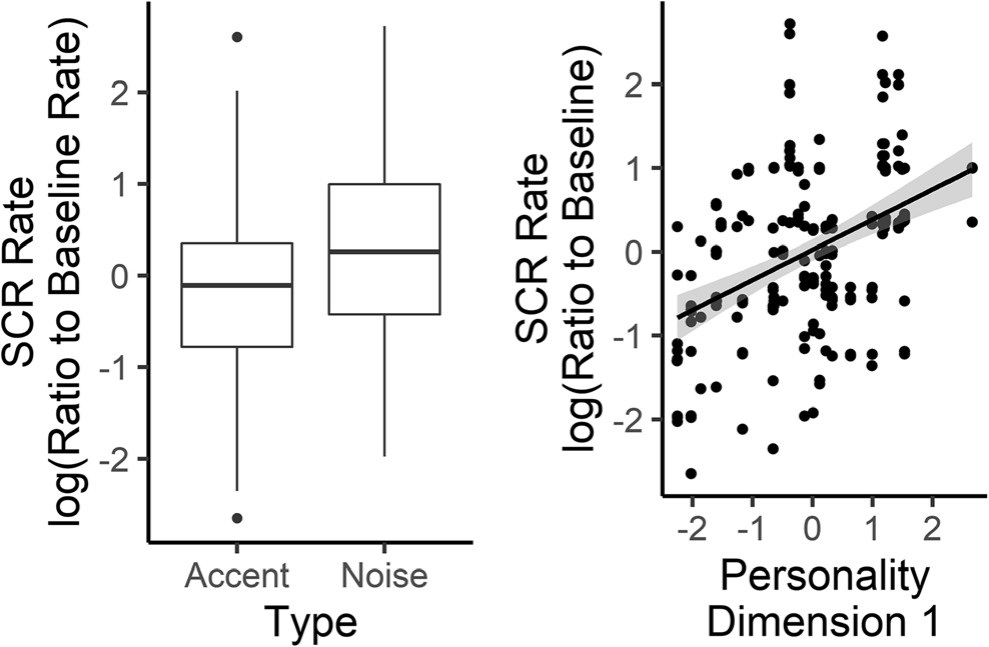
Results of the analysis of rate of nonspecific skin conductance responses (SCR ate). Boxplots show median (black line), Interquartile range (IQR; box), range of ±1.5 × IQR (whiskers), and outliers (dots). In the scatterplot, each symbol represents a single trial for a single participant. Line represents linear regression with shaded 95% CI. Personality Dimension 1 is associated with the Big Five traits of extraversion, agreeableness, and conscientiousness

Participants showed an overall increase in the rate of SCRs compared with baseline, with log ratios averaging 0.33 (*SD* = 0.32, range: −0.51–1.02) . Results of the analysis of the linear model showed only significant contributions of condition, *F*(1, 5.39) = 24.68, *p* = .003, and Personality Dimension 1 (related to extraversion, agreeableness, and conscientiousness), *F*(1, 16.69) = 5.05, *p* = .038, as shown in Fig. 6. These results suggest that listening to stories presented in noise leads to a greater increase in the rate of skin conductance responses than does listening to comparably difficult nonnative accented speech. In addition, individuals who score higher on ratings of extraversion and/or agreeableness are more likely to exhibit a greater increase in their rate of spontaneous skin conductance responses under effortful listening conditions (as compared with relaxed baseline), but this relation is not affected by the source of the effort.

#### Heart period

**Fig. 7:**
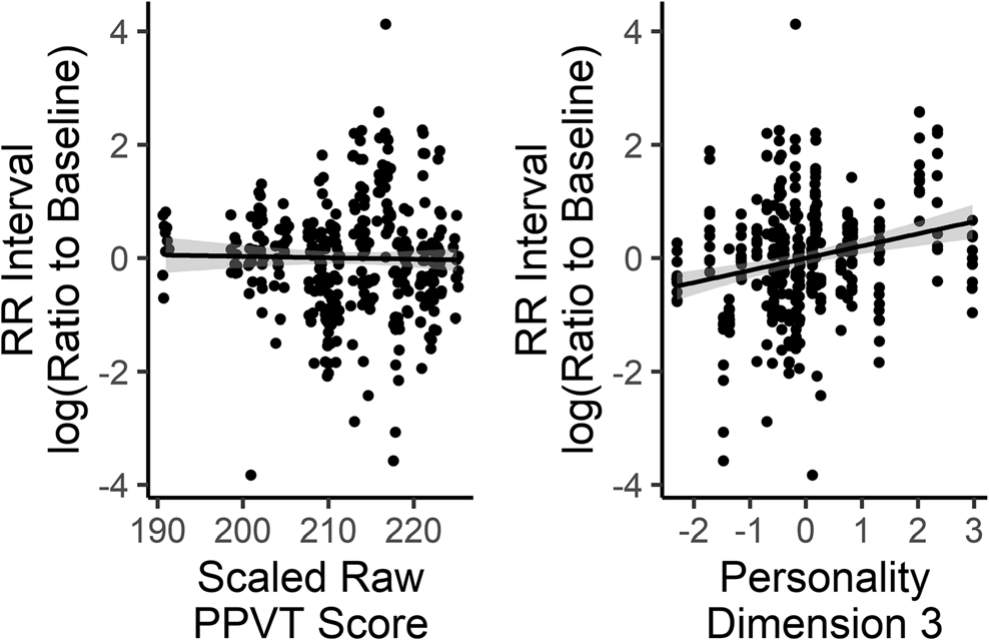
Effects of Vocabulary score (PPVT Raw) and Personality Dimension 3 (neuroticism and conscientiousness) on scaled and centered log ratio of RR interval to baseline (greater positive change indicates greater decrease in heart rate). Each symbol represents a single trial for a single participant. Lines represent linear regression with shaded 95% CI

**Fig. 8:**
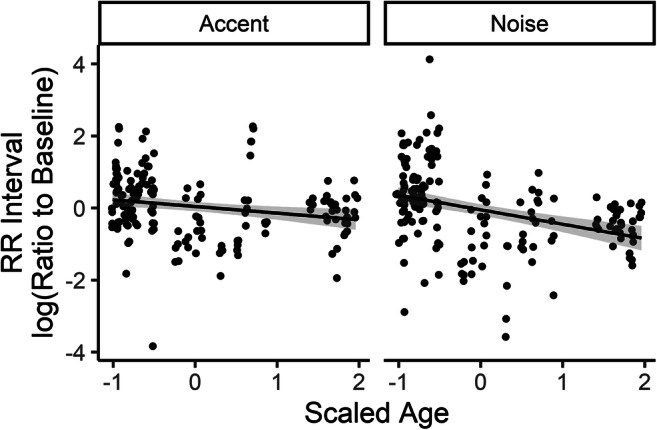
Scaled and centered age versus scaled and centered log ratio of RR interval to baseline in both conditions. Each symbol represents a single trial for a single participant. Lines represent linear regression with shaded 95% CI

**Fig. 9:**
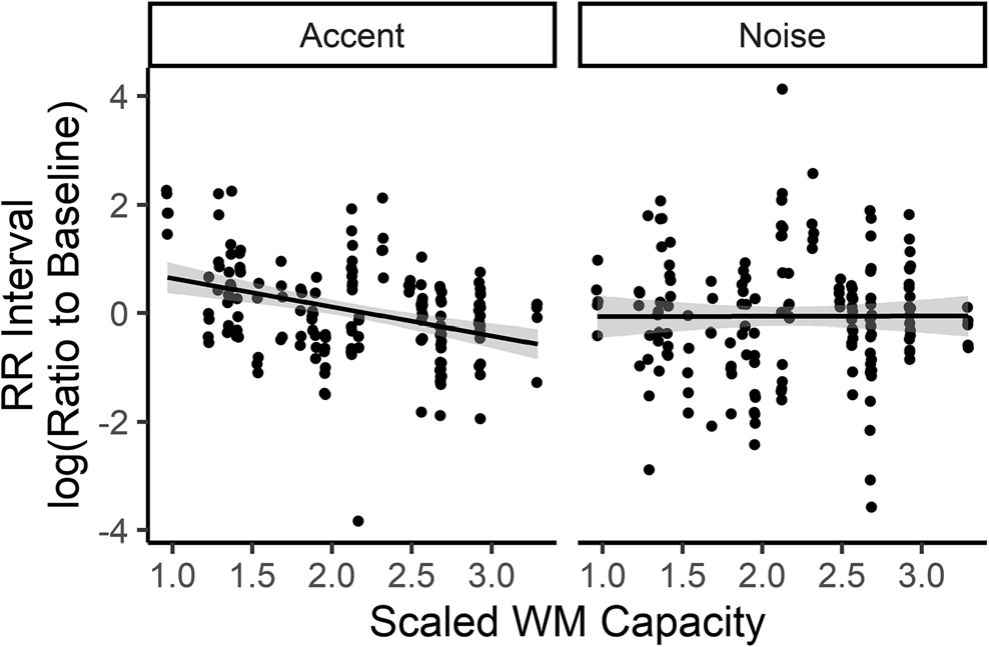
Scaled and centered WM capacity score (*d'* on 2-back task) versus scaled and centered log ratio of RR interval to baseline. Each symbol represents a single trial for a single participant. Lines represent linear regression with shaded 95% CI

Participants showed an overall slight decrease in heart period (indicating a slight increase in heart rate) compared with baseline, with a mean log ratio of −0.006 (*SD* = 0.014, range: −0.043–0.030). This measure did not differ across conditions (accent = −0.063, *SD* = 0.18; noise = −0.065, *SD* =0.16). However, analysis of the linear model showed significant contributions from Personality Dimension 3 (associated with neuroticism and conscientiousness), *F*(1, 22) = 7.01, *p* = .015, and vocabulary, *F*(1, 22) = 5.81, *p* = .025, and a significant interaction between condition and age, *F*(1, 21.87) = 5.54, *p* = .028, and between condition and WM capacity, *F*(1, 21.80) = 7.44, *p* = .012. Figure 7 shows the main effects of the personality dimension and vocabulary on change in heart period versus baseline, while Figs. 8 and 9 show the effect of age and WMC broken down by listening challenge condition, respectively. Note that, although the data points with normalized heart period values close to ±4 are likely outliers (as they appear to correspond to changes in heart period of around ±100 ms, which is very large), re-running the analysis excluding the three participants whose scores lie outside three standard deviations from the group mean results in substantially similar findings: All of the same factors were significant at *p* < .05 that had been with those three participants included, and all that were not were not.

Taken together, these results suggest that individual traits, including personality, age, vocabulary, and working memory capacity, may affect how listeners respond physiologically to challenging listening conditions. Listeners who score higher on scales of neuroticism and conscientiousness show greater increase in heart period (compared with baseline period), representing greater decrease in heart rate, during challenging listening tasks. In contrast, listeners with greater scores on the PPVT test of vocabulary showed somewhat lower heart period (increase in heart rate) on challenging listening tasks. Both age and working memory capacity showed an interaction between heart period and challenge condition. As listener age increased, heart period decreased relative to baseline, but this effect was stronger in the noise condition than in the accented condition. Finally, individuals with greater working memory capacity also show a decrease in heart period (increase in heart rate) relative to baseline when listening to speech that is challenging because it is produced with a nonnative accent, but not when listening to speech that is challenging because it is masked by steady-state noise.

## Discussion

### Performance

Overall, performance on the story comprehension questions was comparable across the noise and accented conditions, which suggests that, by broadly matching word recognition performance across the two listening challenges on an individual listener basis, we were successful in matching the overall difficulty of the experimental listening tasks as well. Nevertheless, individuals who completed the noise condition with higher SNRs performed somewhat better overall on the story comprehension questions, as did those with larger working memory capacity scores. Because SNR values in the noise condition also determined the absolute level of the single talker in the accented condition, such that higher SNR values correspond to higher target talker levels, it is possible that the main effect of SNR reflects individual differences with respect to talker signal level across both conditions, not just to SNR in the noise condition.^4^ Thus, future research on individual differences in listening performance should consider the role of individual differences in response to absolute target signal level as well as SNR in noise-masked conditions.

The observed correlation between story comprehension score and blood volume pulse amplitude (BVPA) is consistent with research suggesting that sympathetic arousal, perhaps especially as reflected in cardiovascular activity, is positively associated with changes in demand on cognitive processing. For example, Iani et al. (2004) measured peripheral vasoconstriction during a visual working memory task and found that pulse amplitude decreased during task performance and was sensitive to differences in task demand. On the other hand, in the present study, other measures of sympathetic arousal, such as skin conductance level (SCL) and skin conductance responses (SCR), showed no such correlations with story comprehension score, suggesting that sympathetic arousal alone is insufficient to account for the present results. Below, we propose that the present results are consistent with the well-established principle of *directional fractionation*, in which some autonomic physiological measures may show an effect of an experimental manipulation while other measures of the same autonomic branch (in this case, the sympathetic) do not (Bradley, 2000; Francis & Oliver, 2018; Lacey, 1967).

### Listening effort

Examination of self-reported effort (NASA TLX Effort subscale) revealed a significant interaction between the condition (noise masking vs. nonnative accented speech) and the SNR variable. Increasing SNR was associated with a decreasing subjective assessment of effort in the noise condition. However, in the accented condition, where the variable “SNR” reflects Target level alone, increasing Target level was associated with an *increasing* rating of subjective effort. This finding lends further support to the idea that, when listeners are asked to assess exerted effort, they may instead report some other property of the experience. It is possible that this other property could be an estimate of how well listeners thought they were doing (Moore & Picou, 2019), or perhaps how hard the task is supposed to be. In support of the second possibility, in the noise condition increasing SNR should make the task seem easier, whereas in the accented condition, perhaps the increasing target level makes it seem as if the speaker is expecting the listener to have more difficulty and therefore is raising their level. It seems less likely that listeners were assessing some aspect of their overall performance, at least with respect to comprehension, even though effort ratings were made after the final set of comprehension questions in each condition. For example, informal examination of the nonsignificant effect of condition on the relationship between SNR and performance (main effect of SNR shown in the left panel of Fig. 3) shows that as SNR or target level increased, participants did objectively better on the story comprehension task in both the noise and the accented condition. Furthermore, subjective effort rating was largely independent of performance. A two-tailed, Pearson’s correlation analysis of effort and actual performance (mean score on the poststory questions) showed no significant correlation, *t*(68) = −1.31, *p* = .19. We interpret this pattern of results as suggesting that listeners may have been incorporating some sense of the overall SNR and/or target level into their estimate of task difficulty, such that higher target levels were perceived as less effortful when they appeared in the presence of noise, but were perceived as more effortful when imposed on nonnative accented speech in the absence of masking noise.

Self-rated effort did not correlate significantly with story comprehension, which might not be expected, except that (a) the phrasing of the rating task explicitly mentions performance (“How hard did you have to work to accomplish your level of performance”) and (b) effort ratings were made immediately after completing the final 10 comprehension questions of the block so listeners might be more cognizant of the evaluation of their performance at that time. On the other hand, listeners did not receive feedback on their performance during the listening task, so it seems unlikely that they would have a very accurate sense of it, Self-rated effort also did not correlate with any of the physiological measures associated with listening effort. These null effects may simply be a consequence of the small number of participants and resulting (presumably) underpowered nature of the study. It could also be related to the fact that effort was assessed only twice, once after each condition. However, the lack of relationship between subjective effort ratings and physiological effort measures is also consistent with recent research suggesting that these two different types of measures may reflect different aspects of listening effort (Francis and Love, 2020; Strand et al., 2018). Further research is needed to better understand what, exactly, listeners are considering when self-reporting effort, but in the present case it seems unlikely to be related to an awareness of actual performance.

#### Electrodermal response, sympathetic arousal, and noise

Measures of electrodermal activity (skin conductance level [SCL] and rate of spontaneous skin conductance responses [SCRs]), which are associated with sympathetic arousal, were both affected more in the noise condition than in the accented condition, albeit in different ways. First, SCR rate was overall higher in the noise condition than the accent condition. In contrast, there was no main effect of condition on SCL. This discrepancy is somewhat unexpected as the two measures (SCL and SCR Rate) were correlated overall (see Table 5). While the physiology underlying these two measures suggests they should correspond with one another at least to a broad degree, the fact that they pattern differently according to listening condition here (as well as in other studies) is consistent with the possibility that the different electrodermal activity responses may reflect autonomic responses to at least partially distinct qualities of stimulation and/or concomitant internal states (Dawson et al., 2007).

Second, in the noise condition, SCL was lower for those with higher pure-tone thresholds. Recall that presentation levels for the stories in both conditions, as well as noise levels in the noise condition, were set individually according to performance on the initial word recognition calibration task. Therefore, listeners with elevated pure-tone thresholds (i.e., decreased hearing sensitivity), who might have performed worse on the initial level calibration task, could consequently have also ended up with higher signal (and lower noise) levels. The resulting across-listener differences in SNR could, in turn, have made the task easier for them, with consequent lowering of arousal as reflected in SCL. Although Best Ear PTA thresholds were not significantly correlated with SNR levels across individuals in the present experiment, *t*(33) = 1.20, *p* = 0.237, *R* = 0.21, this null result could have been due to the small number of participants rather than the absence of a relationship. Further research will be necessary to determine the precise nature of the relationship between hearing levels, SNR, overall sound level, and EDA.

Finally, there was an interaction between Personality Dimension 1 (associated with the Big Five traits of extraversion and agreeableness) and SCR rate, such that, as extraversion and/or agreeableness scores rose so too did the rate of SCR responses. This finding, suggests that more extroverted and/or agreeable listeners exhibited somewhat stronger sympathetic arousability, which is arguably broadly consistent with the idea that people who are more extroverted are more electrodermally “labile” (i.e., exhibit a greater rate of nonspecific and stimulus-related SCRs; Crider, 1993). On the other hand, it may be at odds with the expectation that people who are higher in extraversion should exhibit fewer stimulus-related SCRs under stress, though this, too, may vary depending on the source of the stress (Brumbaugh, Kothuri, Marci, Siefert, & Pfaff, 2013). Taken together, analysis of electrodermal responses suggest that the effects of noise and noise-related effort may be productively investigated through more targeted studies employing skin conductance measures of sympathetic arousal as a dependent measure. However, this relationship must be considered within the context of individual, potentially context-dependent, differences in propensity for sympathetic arousal, a topic apparently in need of significant further research.

#### Heart period

Examination of heart period (inverse of heart rate) showed a number of significant effects. In particular, listeners with greater working memory capacity appear to show a greater magnitude of decrease in heart period (greater increase in heart rate) in response to challenging listening conditions, but possibly only in the accented condition. The fact that the accented condition shows a stronger influence of working memory capacity on listening challenge-related changes in heart period is consistent with the hypothesis that understanding nonnative accented speech may be particularly demanding on working memory capacity while listening to native accented speech is considerably less demanding on working memory processing. Similarly, individuals with larger vocabularies tended to show a greater increase in heart period, though there was no interaction with Condition in this case. Decrease in heart period is related to both sympathetic arousal and withdrawal of parasympathetic activity and has been associated with the engagement of working memory capacities (Backs & Seljos, 1994). The present findings are consistent with an understanding of shorter tonic heart period as reflecting overall greater engagement of working memory in a task. Following this interpretation, it is possible that the two listening challenges differ in terms of the degree to which they place demand on working memory capacity. Listening to a nonnative accent may incur greater demand on working memory capacity than listening to noise-masked speech.

Although previous research suggests that greater cardiovascular arousal may be associated with better performance on memory-demanding tasks (Backs & Seljos, 1994), there was no significant correlation between comprehension task performance and heart period, *t*(348) = 0.72, *p* = 0.471, *R* = 0.039, or between rated effort and heart period, *t*(68) = 1.24, *p* = 0.220, *R* = -0.148. Ultimately, these results suggest that, although listening to nonnative accented speech may impose greater demand on working memory capacity than does listening to noise-masked speech (consistent with the findings of McLaughlin et al., 2018), the greater engagement of working memory capacity did not lead to improved performance or greater self-reported perception of effort, at least on the present tasks and/or for the present participants.

The comprehensive pattern of results observed here is consistent with the interpretation that decreasing heart period may reflect the engagement of working memory. This engagement seems to be stronger in the accented condition (where those with greater WM capacity show a greater decrease in heart period over baseline). Moreover, some of the effect of challenging listening conditions on heart period seems to be mitigated for those with higher vocabulary scores, although this effect does not seem to interact with the Condition (i.e., nonnative vs. noise-masked speech). Nevertheless, these results combine to suggest that both working memory capacity and vocabulary may affect heart period in response to listening challenges, especially those conditions that impose a substantial demand on working memory capacity. Together the present findings support the need for future research on the interaction between working memory capacity, lexical knowledge, and perception of nonnative accented speech. Based on the present results, we suggest that such research may productively employ measures related to heart period as a dependent measure associated with demand on or engagement of working memory and lexical knowledge.

Finally, the degree to which heart period changes from baseline under challenging listening conditions is also affected by personality traits. Heart period decreases more for those who score high on the personality dimension associated with Neuroticism and Conscientiousness. However, these effects do not interact with Condition. As with the findings related to electrodermal activity, these results suggest a need to consider individual differences in physiological responsivity when using physiological measures as indices of listening effort. For example, Dawson et al. (2007) discuss a procedure for normalizing individual participants’ task-related SCL values against the *range* of SCL values obtained at rest (as here) and during a stimulation that evokes a (near) maximal response. It is possible that a parallel normalization procedure for heart period may prove valuable in controlling for differences across individuals in future studies.

#### BVPA and EMG

Neither electromyography of corrugator supercilii nor peripheral capillary dilation (BVPA) showed significant effects for any of the factors of interest. While it is not possible to draw any strong conclusions from this lack of effect, a few tentative observations may be made.

With respect to the BVPA, it is important to note that that BVPA *did* correlate significantly with final score on the tests of story comprehension. Because BVPA is a purely sympathetic response, tonic BVPA may thus serve as an index of overall arousal, attentiveness, or vigilance, or perhaps simply engagement in the listening task. Such an interpretation would be consistent with the original association of cognitive effort with physiological, especially sympathetic arousal. For example, there is a long literature showing an “inverted U-shaped” relationship between arousal, especially sympathetic arousal, and performance on many kinds of cognitive tasks (Aston-Jones & Cohen, 2005; Kahneman, 1973; Lentarowicz, Simpson, & Cohen, 2013; Yerkes & Dodson, 1908), such that as arousal increases so does performance up to some optimal point, beyond which continued increasing arousal impairs performance. In this context, the present results suggest that listeners in this experiment may have tended to be on the left side of this inverted “U”, such that increasing sympathetic arousal (reflected in increasing BVPA values) resulted in better task performance. However, we must note that SCL is also a purely sympathetically innervated response, and also one typically seen as an indicator of overall arousal, attentiveness, and task engagement (Dawson et al., 2007), and SCL nevertheless failed to show a relationship with performance in the present study. Thus, one measure of sympathetic arousal (BVPA) showed a correlation with performance while another (SCL) did not.

It is possible that the contrasting patterns of responsivity exhibited by two exclusively sympathetically-governed response, BVPA and SCL, represents a paradigmatic case of directional fractionation (Bradley, 2000; Lacey, 1967), suggesting the need for further research on the basic relationship between engagement in and performance on effortful listening tasks, as well as on the interaction of both engagement and performance with different measures of sympathetic nervous system arousal. One likely interpretation of such fractionation in the present case is that cardiovascular measures, perhaps especially blood pressure regulation and possibly related peripheral vascular activity, are associated with cognitive processing demands (Duscheck et al., 2009). However, this relationship is not governed by activation of the sympathetic nervous system as a whole. Rather, there may be a specific and probably reciprocal relationship between cardiovascular activity and cognitive processing that has yet to be determined (cf. discussions by Critchley, Eccles, & Garfinkel, 2013; Duschek, Muckenthaler, Werner, & Del Paso, 2009). Future work in this area could focus on identifying the specific neural systems engaged by cognitive processing that may be associated with control of cardiovascular activity, particularly blood pressure regulation. Focusing on implications of the present results, the apparent absence of any effects of task on tonic BVPA in the present study also suggests that, when BVPA is used to assess listening effort, it might be more productively considered in terms of its phasic response pattern to individual stimuli (e.g., Francis et al., 2016) rather than as a tonic measure, as in the present study.

With respect to electromyography, it is possible that the manipulations used in the present study simply did not evoke the degree of affective response typically seen in studies that evaluate corrugator activity. For example, Larsen et al. (2003) used stimulus sets that included images of mutilated bodies, alarm sounds, and written insulting words, along with other lower-valence tokens. It seems reasonable to expect that such high-valence stimuli would evoke a stronger corrugator response than mere displeasure or annoyance at an interfering background noise or effort-inducing nonnative accent. On the other hand, there were still measurable differences from baseline even for the lower-valence tokens in that study. Moreover, corrugator can show a measurable response to something as affectively subtle as the mere awareness that one has made an error on individual trials in a basic cognitive task. Dignath, Berger, Spruit, and van Steenbergen (2019) found a significant increase in corrugator activity following incorrect responses on a Stroop-like task, suggesting that this muscle can reliably indicate even relatively mild, internally-generated affective responses. However, this error-related corrugator response studied by Dignath and colleagues was highly phasic and declined rapidly and even reversed to some degree over the course of about a second after the error was made. It seems unlikely that such a rapidly occurring and declining physiological responses would leave a lasting impression on a tonic measure collected over minutes of listening time as in the present study. Taken together, this evidence of identifiable phasic responses to a variety of aversive stimuli combined with the present lack of an observable tonic effect in corrugator responsivity to listening effort suggests that corrugator may be more useful as an event-related (phasic) measurement of discomfort or displeasure rather than a tonic response to listening effort as was attempted in the present study. For example, future studies could measure corrugator response for nonnative accented speech focusing specifically at time points at which speakers’ pronunciations clearly diverge from native norms. Such divergences may result in increased effort due to the need to map the unfamiliar pronunciation onto a lexical item, which in turn could be reflected in phasic corrugator activation.

### General discussion

A few overarching patterns may be observed across measures in the present results, which, although tentative, may provide useful guidance for future research. First, we observed that SNR, which corresponded to Target level in the accented condition, had opposite effects on self-assessed effort ratings across the two conditions. We interpret this finding as consistent with the growing consensus that, when asked to rate effort levels, listeners may be evaluating different subjective qualities of the task and/or their task-related internal state in response to different kinds of challenges (Picou et al., 2019), and thus listeners’ responses may have been addressing or affected by different properties of the task in the two conditions.

Second, we observed patterns of directional fractionation across different measures. For example, electrodermal responses (SCL and SCR rate) showed the strongest effects of individual differences in the noise condition, exhibiting a dependence on SNR and hearing sensitivity. On the other hand, heart period was most strongly associated with individual differences in working memory capacity and vocabulary, especially in the accented condition. Based on these differences, and despite the potential confound in the present study between hearing thresholds and SNR in the EDA results, this pattern of results across physiological measures and listening challenges suggests that future research might productively be designed to investigate the following more explicit (if still highly speculative) hypotheses: (1) Electrodermal measures seem likely to be more reflective of listening effort-related mental activity under conditions that involve extrinsic noise, or that otherwise induce distraction, annoyance, frustration, or similar affective responses with higher negative valence. However, enthusiasm for this hypothesis may be tempered by the observation that neither of the present listening challenges induced a measurable EMG response in corrugator supercilii, suggesting that if there was any effect of valence, it was too small to be identified in the range activity of this muscle that we were capable of registering. (2) Cardiac measures should prove more successful when measuring listening effort-related mental activity evoked by tasks that increase demand on working memory capacity. However, the strength of this cardiac response, or of the demand evoking it, may be mitigated in individuals with stronger linguistic capabilities, especially vocabulary.

With respect to the design of future research, the present results also highlight the need for care when interpreting physiological results. First, we emphasize the need to distinguish (and decide) between tonic and phasic uses of particular physiological variables. While both EMG and BVPA have been shown to reliably reflect relatively subtle differences in affective response to phasic stimuli in previous research, neither proved sufficiently sensitive when used in the present study in a more tonic manner. Secondly, the correspondence (or lack thereof) between physiological and self-report measures must be interpreted with care. For example, in the present study, SNR affected performance and self-rated effort, but had no effect on physiological measures. The absence of an effect of SNR on physiological measurements should not be taken as suggesting that SNR cannot affect physiological responses, but rather suggests mere that sound level-related effects on physiological response magnitude are negligible within the range of stimulation, and within the range of physiological response magnitudes measured, used here. Finally, the fact that some of the measures used here appear to interact with individual differences in personality traits suggests a need for further research into the interaction between trait-level characteristics and assessments of listening effort, both from a theoretical and from a methodological perspective.

## Conclusion

Our results suggest that the psychological construct of listening effort may be differentiable according to the relative dependence of speech perception on various cognitive processes, with these conclusions deriving from the outcomes of different groupings of subjective and psychophysiological measurements. On the one hand, the effort of understanding nonnative accented speech is more closely associated with demand on working memory capacity than is the effort of understanding native-accented speech masked by steady-state noise, as suggested by the greater role played by heart period and working memory capacity in the accented condition as compared with the noise condition. On the other hand, the noise-masked condition appears to have induced a stronger negative affective response, measured in terms of greater involvement of electrodermal activity and therefore perhaps reflecting greater distraction or annoyance, than did the nonnative accented condition. From a broader perspective, the present results also add to the growing body of work suggesting that physiological measures of effort provide information that supplements, rather than replaces, evaluations of performance and subjective ratings of effort.

## Footnotes

1 Note that the stories originally assigned as practice by Fossett et al. (2004) were treated equivalently to the others—one was used here as a practice story, but this decision was made only on the basis of pilot test group performance only.

2 It should be noted that a total of 30 trials is considered low for these methods and may not be sufficient to identify definitive SNR levels for these performance targets (Levitt, 1971). However, the goal of this procedure was simply to identify an SNR at which it was reasonably likely that speech comprehension would be matched across noise-masked and nonnative accented conditions for each individual listener.

3 One of the first few participants received a target level of 53.3 dB SPL and a noise level of 63.7 dB (SNR of −10.4 dB) before the caps were instituted. This participant was the only one with level values outside the capped range.

4 Note that target levels ranged from 53.3 to 59.6 dB SPL (mean of 56.8 dB), so it is possible that participants with lower SNR values also found the nonnative accented speech slightly more challenging to hear as well.
